# Quantitative Structural Brain Magnetic Resonance Imaging Analyses: Methodological Overview and Application to Rett Syndrome

**DOI:** 10.3389/fnins.2022.835964

**Published:** 2022-04-05

**Authors:** Tadashi Shiohama, Keita Tsujimura

**Affiliations:** ^1^Department of Pediatrics, Chiba University Hospital, Chiba, Japan; ^2^Group of Brain Function and Development, Nagoya University Neuroscience Institute of the Graduate School of Science, Nagoya, Japan; ^3^Research Unit for Developmental Disorders, Institute for Advanced Research, Nagoya University, Nagoya, Japan; ^4^Department of Radiology, Harvard Medical School, Boston, MA, United States; ^5^Athinoula A. Martinos Center for Biomedical Imaging, Massachusetts General Hospital, Charlestown, MA, United States

**Keywords:** quantitative analysis, voxel based morphometry, surface based morphometry, diffusion-weighted MRI tractography, rett syndrome (RTT)

## Abstract

Congenital genetic disorders often present with neurological manifestations such as neurodevelopmental disorders, motor developmental retardation, epilepsy, and involuntary movement. Through qualitative morphometric evaluation of neuroimaging studies, remarkable structural abnormalities, such as lissencephaly, polymicrogyria, white matter lesions, and cortical tubers, have been identified in these disorders, while no structural abnormalities were identified in clinical settings in a large population. Recent advances in data analysis programs have led to significant progress in the quantitative analysis of anatomical structural magnetic resonance imaging (MRI) and diffusion-weighted MRI tractography, and these approaches have been used to investigate psychological and congenital genetic disorders. Evaluation of morphometric brain characteristics may contribute to the identification of neuroimaging biomarkers for early diagnosis and response evaluation in patients with congenital genetic diseases. This mini-review focuses on the methodologies and attempts employed to study Rett syndrome using quantitative structural brain MRI analyses, including voxel- and surface-based morphometry and diffusion-weighted MRI tractography. The mini-review aims to deepen our understanding of how neuroimaging studies are used to examine congenital genetic disorders.

## Introduction

Congenital genetic disorders often present with neurological manifestations. Such as neurodevelopmental disorders, motor developmental retardation, epilepsy, and involuntary movement. In both clinical practice and research, multiple neuroimaging modalities are used to identify the lesions responsible for each symptom or syndrome. Magnetic resonance imaging (MRI) is widely recognized as the most helpful modality for examining human brain structures *in vivo* because of its high reproducibility, high spatial resolution, and low invasiveness.

Approaches for evaluating MRI can be divided into qualitative and quantitative analyses. Through qualitative morphometric evaluation, remarkable structural abnormalities, such as lissencephaly, polymicrogyria, agenesis of the corpus callosum, and lesions with abnormal signal intensities, can be identified in some patients; however, in clinical settings, no structural abnormalities are identified in a large population of patients with congenital genetic disorders, even in patients with definitive neurological sequelae. Additionally, the interobserver reproducibility of qualitative assessments of cerebral atrophy is less than 50% (Pasquier et al., 1996; Scheltens et al., 1997); therefore, there are potential benefits to attempting automatic quantitative analyses of MRI, even in cases with visually identified cerebral atrophy.

Recent advances in data analysis programs have led to significant progress in the quantitative analyses of structural MRI images, such as three-dimensional T1-weighted images (Frackowiak et al., 1997; Zijdenbos et al., 2002; Fischl, 2012; Jenkinson et al., 2012; Djamanakova et al., 2014; Manjón and Coupé, 2016) and diffusion-weighted MRI tractography (Wang et al., 2007; Berman, 2009; Webster and Descoteaux, 2015; Norton et al., 2017; Yeh, 2020), as well as MR spectroscopy (Takanashi, 2015), arterial spin labeling (Parkes and Tofts, 2002), and functional MRI (Biswal, 2012). These approaches have been used to investigate healthy volunteers, psychological disorders, and congenital genetic disorders such as Down syndrome (Hamner et al., 2018; Levman et al., 2019; Brown et al., 2021), Turner syndrome (Zhao and Gong, 2017), and Rett syndrome (Casanova et al., 1991; Murakami et al., 1992; Reiss et al., 1993; Subramaniam et al., 1997; Carter et al., 2008; Mahmood et al., 2010; Oishi et al., 2013; Shiohama et al., 2019).

Approximately 6,000 single-gene disorders are listed in public databases, such as Online Mendelian Inheritance in Man (OMIM),^1^ which also covers neuroradiographic findings in most congenital genetic diseases. Both qualitative and quantitative evaluation of the morphometric characteristics of the brain in congenital genetic disorders leads to basic and clinical benefits. From a clinical perspective, brain phenotyping may contribute to identifying neuroimaging biomarkers for early diagnosis and response evaluation in patients with congenital diseases. Fundamentally, brain phenotyping contributes to revealing the function of single genes in the human brain structure at an individual level. Because there are several differences in gyral and sulcal structures and transcriptional expression between the human and mouse brain (Hodge et al., 2019), neuroimaging studies in patients with pathogenic variants could provide important information beyond that provided by transgenic mouse models.

Since the late 2000s, expanded indications for next-generation sequencing has led to a paradigm shift in the leading diagnostic approach in undiagnosed congenital disorders from clinical physiological findings to comprehensive genetic testing. The genetic variants leading to diagnosis expanded the previously known clinical spectrum of congenital disorders. Subsequently, deep phenotyping using automatic program analyses has attracted the attention of many morphologists in the redefinition of the clinical spectrum of previously known congenital disorders, which was referred to as next-generation phenotyping (van der Donk et al., 2019). This mini-review focuses on methodologies and attempts employed to understand Rett syndrome using quantitative structural brain MRI analyses, including voxel- and surface-based morphometry and diffusion-weighted MRI tractography. The mini-review aims to deepen our understanding of how neuroimaging studies are used to examine congenital genetic disorders.

## Quantitative Brain Magnetic Resonance Imaging Analysis

### Brain Morphological Development

In typical human brain development, uniformity of cortical thickness, myelination, dendritic arborization, and remodeling and pruning of synapses progress over time from the fetal period, leading to the maturation of cortical sulcal/gyral patterns between gestational age of 16 weeks and 1 month after birth (Armstrong et al., 1995; de Graaf-Peters and Hadders-Algra, 2006; Gilmore et al., 2018; Barkovich and Raybaud, 2019; Ouyang et al., 2019). The overall brain size increases, reaching approximately 90% of the adult volume from birth to 2 years of age (Giedd and Rapoport, 2010). The volume of the cortical gray matter (CGM) and white matter (WM) has been shown to increase by 4.6 and 1.9 times, respectively, from gestational ages of 30 to 40 weeks (Moeskops et al., 2015). The volume of the CGM and WM increases 108–149 and 11%, and 14–19 and 19% from birth to age 1 year, and from age 1 year to age 2 years, respectively (Gilmore et al., 2018). The volume of the CGM slightly increases during childhood and decreases during adolescence, while the WM continuously increases in size until approximately age 30 years (Courchesne et al., 2000; Matsuzawa et al., 2001; Gilmore et al., 2018). In contrast, cortical thickness and the CGM surface area exhibit negative and positive correlations with participant age, respectively (Levman et al., 2017). Surface area expansion is regionally heterogeneous across the brain, with dominance of the lateral frontal, lateral parietal, and occipital cortex (Gilmore et al., 2018). Most commissural, projection, limbic, and associative bundles can be identified by diffusion-weighted MRI tractography, even in early infants (Dubois et al., 2014).

Anatomical structures have been recognized to be tightly associated with brain functions, which serves as the scientific foundation for brain morphological studies in human disorders. In a meta-analysis, the intelligence quotient in adults was positively associated with cortical volumes (Pietschnig et al., 2015). Cortical thickness of the prefrontal and posterior temporal cortex (Narr et al., 2007) in a study with healthy adults and the volume of the orbitofrontal cortex and cingulate gyrus in a study with young participants (Frangou et al., 2004) was also reported to be associated with intelligence quotient.

### Anatomical Structural Morphometry

Three-dimensional T1-weighted gradient-echo images (3D T1WI) as the sole or combination with 3D fluid-attenuated inversion recovery images (FLAIR) are commonly employed for anatomical structural brain morphometric studies. The sequence varied between MRI scanner manufacturers as follows: IR-SPGR for GE, MP-RAGE for Siemens, and IR-TFE and MP-RAGE for Philips. When adapting any sequences, sagittal acquisition has the advantage of saving acquisition time, and voxel sizes smaller than 1 mm are required for notable resolution.

The regions of interest (ROIs) approach using manual tracing and distance measurements between anatomical landmarks was the classical method for brain morphometry analysis. This manual method has an advantage in that measurements are simply acquired, even without analytic programs or 3D structural MRI; however, it has many disadvantages, such as requiring substantial work to plot the ROIs, intra- and inter-observer bias, and limited detection capability limited to the ROIs. Therefore, most brain morphologic studies use automatic analytic programs.

Frequently used programs to evaluate brain anatomical structures include FreeSurfer^2^ (Fischl, 2012), CIVET^3^ (Zijdenbos et al., 2002), FMRIB Software Library (FSL)^4^ (Jenkinson et al., 2012), Statistical Parametric Mapping (SPM)^5^ (Frackowiak et al., 1997), VolBrain^6^ (Manjón and Coupé, 2016), and MRIcloud^7^ (Djamanakova et al., 2014). These programs comprehensively evaluate multiple measurements over the whole brain with extremely high reproducibility, according to mathematical algorithms.

Most programs calculate both voxel-based morphometry (VBM) (Ashburner and Friston, 2000) and surfaced-based morphometry (SBM) (Fischl and Dale, 2000). Using VBM, the regional volumes of the CGM, WM, GM, subcortical GM, cerebrum, brainstem, and cerebral ventricles are obtained; however, measurements of cortical areas and thicknesses, and cortical sulcal/gyral patterns cannot be obtained.

Cortical surfaces can be modeled using cortical thickness, area, volume, and curvature measurements (Toro et al., 2008; Schaer et al., 2012) in each brain region using SBM (Fischl and Dale, 2000). SBM has a disadvantage in that regions other than the CGM are not covered at all. The measurements of each anatomical region are calculated from surface data by being parcellated into anatomical standard atlases including Brodmann’s brain map (Zilles and Amunts, 2010), Desikan–Killiany atlas (aparc atlas) (Fischl et al., 2004), Destrieux atlas (aparc.a2009s atlas) (Desikan et al., 2006), and Desikan–Killiany–Tourville atlas (aparc.DKT40 atlas) (Klein and Tourville, 2012). Because the priority among these atlases for parcelation in human brains is controversial, the parcelation atlas employed varies between studies. The values of the cortical thickness of over 40,000 vertices were also obtained from the surface data of each hemisphere and could be utilized for visualizing statistical analyses on the cortical map.

Several curvature measurements have been proposed for quantifying local gyral and sulcal structures including the local gyrification index (Schaer et al., 2012), folding index (Toro et al., 2008), intrinsic curvature index (Pienaar et al., 2008), mean curvature (Pienaar et al., 2008), and Gaussian curvature (Pienaar et al., 2008). Shallow gyri and sulci pattern have been associated with dysfunction of the cortical area and its projection neural fibers (Im and Grant, 2019); however, physiological interpretation of the profiling data of the local curvature measurements is puzzling because multiple covariates, such as age, sex, perinatal events, and comorbidities, contribute to the fine cortical curvature.

Cortical thickness, surface area, cortical volume, and curvature of each region are statistically evaluated as a raw value, the laterality index (Springer et al., 1999), the laterality score (Kelley et al., 2005) or the asymmetry index (Fischl, 2012; Levman et al., 2017).

Some programs, such as FreeSurfer, have an optional tool to add manual interventions to correct the pial surface or voxel segmentation after automatic segmentation. Manual intervention could be attempted for automatic analysis procedures with minor errors instead of removing them from subsequent statistical analyses. However, even when manual edits are performed according to a strictly defined protocol, the improvement in the quality of regional segmentation is limited (Beelen et al., 2020; Monereo-Sánchez et al., 2021).

### Diffusion-Weighted Magnetic Resonance Imaging Tractography

Diffusion-weighted imaging (DWI) is an MRI sequence with signal contrast based on the micrometric movement of water molecules within a voxel of tissue. DWI techniques can be employed for fiber tractography and simple 2D mapping, which is clinically used to detect cellular edema in the acute phase of brain ischemia edema or acute encephalopathy. Fiber tractography is useful for visually identifying communication fibers and association fibers both in living patients and postmortem brains (Vasung et al., 2019). The neuronal fibrillar structure consists of coherently aligned axons surrounded by glial cells. Among these components, the cell membrane leads to the anisotropy of molecular diffusion predominantly in MR tractography-derived fibers rather than myelin, axonal transport, microtubules, or neurofilaments (Hagmann et al., 2006).

Several tractography algorithms, such as fiber assignment by continuous tracking (FACT) (Mori et al., 1999), probabilistic diffusion tractography (Behrens et al., 2007), diffusion spectrum imaging tracking (Wedeen et al., 2008), constrained spherical deconvolution (CSD) (Tournier et al., 2012), diffusion tensor imaging (DTI) (Pajevic and Pierpaoli, 1999; Berman, 2009), and high angular resolution diffusion imaging (HARDI) (Webster and Descoteaux, 2015) have been proposed to distinguish fiber tractography from diffusion MRI. In particular, DTI and HARDI are utilized for ROI-oriented tractography, and TrackVis^8^ (Wang et al., 2007), DSI studio^9^ (Yeh, 2020), and slicerDMRI^10^ (Norton et al., 2017) are frequently employed.

On the DTI (Pajevic and Pierpaoli, 1999; Berman, 2009), the principal direction and fractional anisotropy (FA) in the diffusion of water molecules are shown as an ellipsoid for each voxel according to the diffusion tensor model. On the HARDI (Webster and Descoteaux, 2015), fiber tractography is reconstructed according to the spherical function model (e.g., an orientation distribution function or ensemble average propagator field). Unlike DTI, HARDI can account for multiple crossing fibers within complex fiber architecture such as the centrum semiovale, pons, and cerebellum (Hagmann et al., 2006; Re et al., 2016). The HARDI requires a longer acquisition time than traditional DTI imaging, which has fewer diffusion-weighted volumes.

**FIGURE 1:**
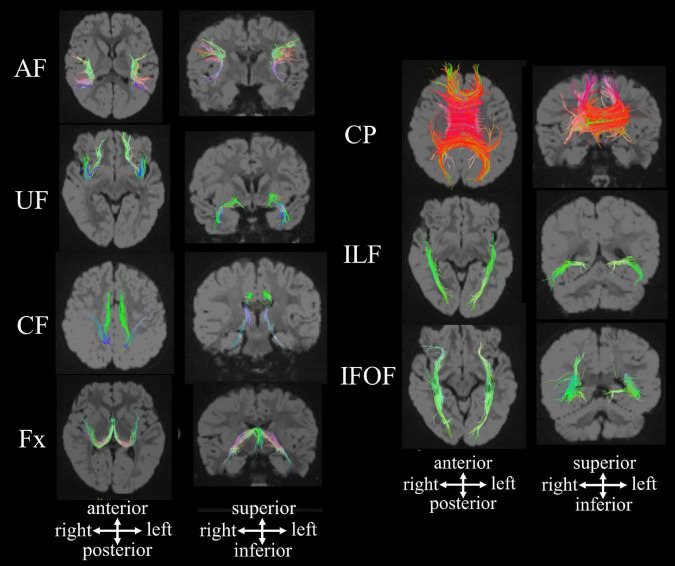
HARDI-based tractography showing the callosal pathway and long association fibers in a 2.5-year-old neurotypical girl [reproduced with permission to reuse after minor revisions (Shiohama et al., 2020)]. AF, arcuate fasciculus; CF, cingulum fasciculus; CP, callosal pathway; Fx, fornix; IFOF, inferior fronto-occipital fasciculus; ILF inferior longitudinal fasciculus; UF, uncinate fasciculus.

Specific fibers of interest can be identified by manually guiding regions of interest (ROIs) on non-diffusion-weighted (b0) images or color FA maps according to anatomic and tractography atlases (Catani and Thiebaut de Schotten, 2008; Mori and Tournier, 2013). In our pipeline, we quantitatively analyzed the mean length, volumes, fractional anisotropy (FA) value, and apparent diffusion coefficient (ADC) value in 13 fibers, including the callosal pathway (CP), bilateral association fibers (arcuate fasciculus [AF], uncinate fasciculus [UF], cingulum fasciculus [CF], fornix [Fx], inferior longitudinal fasciculus (ILF), and inferior fronto-occipital fasciculus (IFOF) (Figure 1; Shiohama et al., 2020).

The FA value is positively related to the degree of directivity of the axon, myelin sheath, and microtubules. Water molecules in brain tissue will move in multiple directions, regardless of whether they are in the white matter, gray matter, or CSF (Campbell and Pike, 2014; Ouyang et al., 2019). Lower FA values indicate that water molecules are more isotropically diffused in the given environment. FA values are close to one in locations where water molecules move disproportionally in one direction (e.g., white matter) (Pecheva et al., 2018). After birth, the FA value in fibers sharply increases prior to the myelination period, which is associated with the maturation of the axonal membrane and increased axonal diameter, microtubule-related protein, and oligodendrocyte (Pecheva et al., 2018). The ADC is higher in locations where water molecules freely diffuse (e.g., CSF), depending on anisotropy, unlike the FA value. The ADC value in fibers drops rapidly during infancy and toddlerhood, which is associated with myelination and axonal pruning, and subsequently plateaus until adulthood (Pasternak et al., 2009; Pecheva et al., 2018). The combination of low FA and high ADC values appears in vasogenic edema, glial scarring, demyelination, and the neonatal brain (Sagar and Grant, 2006; Löbel et al., 2009; Roosendaal et al., 2009; Pecheva et al., 2018). In contrast, a combination of high FA and low ADC values is observed in the white matter of macrocephalic syndrome, which is associated with reduced free water in the intercellular space due to increased axonal density (Oikawa et al., 2015; Shiohama et al., 2020).

Regarding approaches other than ROI-orientated fiber tractography, tract-based spatial statistics (TBSS) (Smith et al., 2006) and tracts constrained by underlying anatomy (TRACULA) (Yendiki et al., 2011) have been attempted to comprehensively evaluate white matter pathways. TBSS (Smith et al., 2006) is a VBM-style method that visualizes FA values over the whole brain without pre-specification of the tracts of interest. After tuning non-linear registration and creating the group mean FA map, each subject’s FA data were projected onto the mean FA skeleton, and subsequently, voxel-wise statistic al analysis was performed across subjects on skeleton-space FA (Smith et al., 2006). TRACULA (Yendiki et al., 2011) is a program provided as a tool for FreeSurfer to automatically identify long association fibers from DWI and T1-weighted images.

### Attempting of Quantitative Structural Brain Magnetic Resonance Imaging for Rett Syndrome

Rett syndrome (RTT; OMIM #312750) is a neurodevelopmental disorder characterized by autistic features, acquired microcephaly, loss of purposeful hand skills, habitual hand clapping, and autonomic dysfunction (Neul et al., 2010; Singh and Santosh, 2018). Typical RTT patients present with a severe decline in global development, decreased head circumference, and the emergence of epilepsy after normal development during infantile periods (Neul et al., 2010; Krishnaraj et al., 2017). This regressive pattern of neurodevelopment in RTT has motivated many studies to search for biomarkers for early diagnosis and intervention of RTT (Singh and Santosh, 2018). To identify specific biomarkers, quantitative structural MRI studies have been carried out (Casanova et al., 1991; Murakami et al., 1992; Reiss et al., 1993; Subramaniam et al., 1997; Carter et al., 2008; Mahmood et al., 2010; Oishi et al., 2013; Shiohama et al., 2019; Table 1).

**TABLE 1:** Quantitative structural brain magnetic resonance imaging studies in Rett syndrome.

Authors	Subjects, N	Age, average (years)	Methods	Findings
**T1-weighted images**				
Casanova et al. (1991)	8	5.3	Manual segmentation	Decreased area of the whole brain hemisphere and bilateral caudate nucleus
Murakami et al. (1992)	13	12.0	Manual segmentation	Decreased area of the cerebrum, basal ganglia, cerebellum, corpus callosum, and brainstem
Reiss et al. (1993)	11	10.1	Manual segmentation	Decreased volume in the cerebrum (dominantly in the GM and frontal lobe), caudate nucleus, and midbrain
Subramaniam et al. (1997)	20	9.7	Manual segmentation	Global reduction in GM and WM volumes except for the pons
Carter et al. (2008)	23	8.6	ABM, VBM	Global reduction in GM and WM volumes with a dominance of the dorsal parietal GM
Shiohama et al. (2019)	7	5.2	VBM, SBM	Decreased volumes in the cerebellum
**Diffusion-weighted images**				
Mahmood et al. (2010)	32	5.5	DTI	Reduced FA in the corpus callosum and external capsule
Oishi et al. (2013)	9	Not described	TBSS	Reduced FA in the left peripheral WM area and tract and the bilateral cingulum

*ABM, atlas-based morphometry; DTI, diffusion tensor imaging; FA, fractional anisotropy; GM, gray matter; SBM, surface-based morphometry; TBSS, tract-based spatial statistics; VBM, voxel-based morphometry; WM, white matter.*

Based on anatomical structural morphometry using T1-weighted images, decreased volumes in the cerebrum (Casanova et al., 1991; Murakami et al., 1992; Reiss et al., 1993; Subramaniam et al., 1997; Carter et al., 2008), basal ganglia (Casanova et al., 1991; Murakami et al., 1992; Reiss et al., 1993), cerebellum (Casanova et al., 1991; Murakami et al., 1992; Shiohama et al., 2019), corpus callosum (Murakami et al., 1992), and brainstem (Murakami et al., 1992; Reiss et al., 1993) have been identified in cases of RTT. Some reports have also noted the dominance of the gray matter in cerebral atrophy (Reiss et al., 1993; Carter et al., 2008) and a decrease in the volume of the cerebellum with age-dependent disease advancement and progression (Murakami et al., 1992; Shiohama et al., 2019). In diffusion-weighted MRI tractography studies, reduced FA in parts of white matter regions (Oishi et al., 2013), the corpus callosum (Mahmood et al., 2010), cingulum (Oishi et al., 2013), and external capsule (Mahmood et al., 2010) has been reported. Although the cerebellar volume may be a potential neuroimaging biomarker of RTT in early infant atrophy, specific brain morphological characteristics of RTT have not been identified, except for non-specific atrophic findings in structural MRI measurements.

## Discussion

### Relevant Issues on Quantitative Brain Magnetic Resonance Imaging Studies to Be Addressed for Evaluating Congenital Genetic Disorders

Most brain morphometric studies of congenital genetic disorders employ two-group comparisons of values calculated from MRI images in multiple patients in a single institution and age- and sex-matched neurotypical controls. Although automatic programs successfully work with MRI images scanned in the clinical setting, attempting brain morphometry for each patient in daily medical care has significant hurdles. For example, automatic brain morphometry analysis requires a long time (e.g., the recon-all program takes 4–5 h per image on the latest version of FreeSurfer), which limits the addition of brain morphometric analyses to routine work.

Approaches using anatomical structural MRI and diffusion-weighted MRI tractography also have limitations themselves. Brain measurements have multiple covariates, such as sex, age at scan, gestational age, comorbidities, and the presence of specific diseases. In addition, there is a critical measurement bias due to differences in MRI scanners (Fortin et al., 2018; Vogelbacher et al., 2018) and analysis software (Lindquist, 2020). Several methods, including visualization of inter-scanner effects on cortical maps (Pardoe et al., 2008), phantom-based scaling correction (Gunter et al., 2009), traveler subjects (Shinohara et al., 2017), and statistical approaches such as ComBat harmonization methods (Maikusa et al., 2021) have been used to control for inter-scanner bias in anatomical structural MRI, while there is no established method to control for the inter-scanner effect in diffusion-weighted MRI tractography.

Concerning anatomical structural MRI, most programs were optimized for brain images in participants over 6 years of age. The rate of segmentation failure is substantially higher for participants aged <8 months, and its reliability is reasonable for participants aged ≥8 months (Levman et al., 2017), at which point myelination contrast patterns have inverted into the mature pattern. We also need to pay attention to the fact that regions on atlases of these anatomical structural and functional regions do not correspond exactly to each other. The left middle and inferior occipital cortices are involved in functions of the secondary visual area identified histologically and, at the same time, overlap with several functionally identified areas, including the human motion-sensitive middle temporal area (hMT/V5) (Thiebaut de Schotten et al., 2014), lateral occipital area (LO) (Thiebaut de Schotten et al., 2014; Bona et al., 2015), occipital face area (OFA) (Gauthier et al., 1999; Bona et al., 2015), and cortical area V8 (color center) (Logothetis, 1999).

Regarding diffusion-weighted MRI tractography, we must consider that reconstructed fibers are very sensitive to the details of tractography algorithms, which can lead to significant false positive and false negative tracking results (Campbell and Pike, 2014).

### Novel Approaches on Quantitative Brain Magnetic Resonance Imaging Studies for the Infant Population

As mentioned above, the widely recognized programs such as FreeSufer, CIVET, and SPM have a common limitation that they are not optimized for MRI imaging of infants, primarily because myelination contrast patterns invert the general pattern from the infant to the toddler period. The reduced tissue contrast, large within-tissue variation, and regional heterogeneity in the infantile brain MRI disturbed a typical pipeline for the adult brain MRI (Li et al., 2019). For this issue, Infant FreeSurfer (de Macedo Rodrigues et al., 2015; Zöllei et al., 2020) [a modified pipeline of FreeSurfer (Fischl, 2012)], a modified pipeline of CIVET (Kim et al., 2016), iBEAT (Dai et al., 2013), and other infant-specific pipelines (Gousias et al., 2008; Leroy et al., 2011; Li et al., 2014, 2015; Makropoulos et al., 2018) have been proposed as optimized programs for infantile participants of 0–2 years old. Based on the steps optimized for the infantile brain MRI (such as image preprocessing, tissue segmentation, image reregistration, regions of interest labeling, topology correction, and surface reconstruction), spatiotemporal cortical surface atlas (Li et al., 2015), and longitudinal volumetric atlas (Zhang et al., 2016) was generated.

Furthermore, challenging attempts using artificial intelligence-based analysis tools have recently been introduced to analyze the MRI images in the infant population (reviewed by Li et al., 2019; Mostapha and Styner, 2019). For example, artificial intelligence-based approaches have been carried out to extract the intracranial volume (Khalili et al., 2019), tissue brain segmentation (Zhang et al., 2015; Moeskops et al., 2016; Nie et al., 2016; Wang et al., 2018; Ding et al., 2020), topology correction (Hao et al., 2016), and FA map analysis (Saha et al., 2020) in the infant population, including early preterm infants.

## Conclusion

This mini-review focused on anatomical structural MRI and diffusion-weighted MRI tractography. These approaches have revealed characteristic findings in patients with congenital genetic diseases through comparisons with neurotypical controls; however, there are significant hurdles (e.g., long time for quantitative analysis, and variety of MRI scanners, acquisition sequences, and analysis pipelines) to overcome in attempting them individually in the clinical setting.

## Author Contributions

TS was responsible for the study design. Both authors wrote/edited the manuscript.

## Conflict of Interest

The authors declare that the research was conducted in the absence of any commercial or financial relationships that could be construed as a potential conflict of interest.

## Publisher’s Note

All claims expressed in this article are solely those of the authors and do not necessarily represent those of their affiliated organizations, or those of the publisher, the editors and the reviewers. Any product that may be evaluated in this article, or claim that may be made by its manufacturer, is not guaranteed or endorsed by the publisher.

## Footnotes

1
https://www.omim.org/statistics/geneMap

2
https://surfer.nmr.mgh.harvard.edu/fswiki/

3
http://www.bic.mni.mcgill.ca/ServicesSoftware/CIVET

4
https://fsl.fmrib.ox.ac.uk/fsl/fslwiki

5
https://www.fil.ion.ucl.ac.uk/spm/software/spm12/

6
https://volbrain.upv.es/

7
https://braingps.mricloud.org/home/

8
http://www.trackvis.org/

9
http://dsi-studio.labsolver.org/

10
http://dmri.slicer.org/
